# In vitro toxicological characterization of two arsenosugars and their metabolites

**DOI:** 10.1002/mnfr.201200821

**Published:** 2013-04-08

**Authors:** Larissa Leffers, Franziska Ebert, Mojtaba S Taleshi, Kevin A Francesconi, Tanja Schwerdtle

**Affiliations:** 1 Graduate School of Chemistry, University of MünsterMünster, Germany; 2 Institute of Food Chemistry, University of MünsterMünster, Germany; 3 Institute of Chemistry – Analytical Chemistry, University of GrazGraz, Austria

**Keywords:** Arsenosugars, Cellular and intestinal bioavailability, Cellular toxicity, Genotoxicity, Marine food

## Abstract

**Scope:**

In their recently published Scientific Opinion on Arsenic in Food, the European Food Safety Authority concluded that a risk assessment for arsenosugars is currently not possible, largely because of the lack of relevant toxicological data. To address this issue, we carried out a toxicological in vitro characterization of two arsenosugars and six arsenosugar metabolites.

**Methods and results:**

The highly pure synthesized arsenosugars, DMA^V^-sugar-glycerol and DMA^V^-sugar-sulfate, investigated in this study, as well as four metabolites, oxo-dimethylarsenoacetic acid (oxo-DMAA^V^), oxo-dimethylarsenoethanol (oxo-DMAE^V^), thio-DMAA^V^ and thio-DMAE^V^, exerted neither cytotoxicity nor genotoxicity up to 500 μM exposure in cultured human bladder cells. However, two arsenosugar metabolites, namely dimethyl-arsinic acid (DMA^V^) and thio-dimethylarsinic acid (thio-DMA^V^), were toxic to the cells; thio-DMA^V^ was even slightly more cytotoxic than arsenite. Additionally, intestinal bioavailability of the arsenosugars was assessed applying the Caco-2 intestinal barrier model. The observed low, but significant transfer rates of the arsenosugars across the barrier model provide further evidence that arsenosugars are intestinally bioavailable.

**Conclusion:**

In a cellular system that metabolizes arsenosugars, cellular toxicity likely arises. Thus, in strong contrast to arsenobetaine, arsenosugars cannot be categorized as nontoxic for humans and a risk to human health cannot be excluded.

## 1 Introduction

Inorganic arsenic is a well-documented human carcinogen after both oral exposure and inhalation. While environmentally relevant arsenic species include inorganic as well as organic arsenicals, elevated cancer incidences have been attributed to inorganic arsenic, and arsenic research has focused on identifying the underlying mechanisms [1–3].

In the general population, diet is the primary source of arsenic intake [4, 5]. Whereas total arsenic content of terrestrial foods is generally low, foods of marine origin usually have a much higher (100-fold) total arsenic content, and this arsenic is mostly present in organic forms [4–6].

Among the water-soluble organic arsenicals identified in marine food, arsenobetaine, which predominates in fish and crustaceans [7], and arsenosugars are the most widespread compounds. Arsenosugars are the major arsenical constituents of marine algae (typically 2–50 mg arsenic/kg dry mass) [8, 9]. They are also present at significant concentrations in animals feeding on algae (e.g. mussels and oysters; typically 0.2–5 mg/kg dry mass) and in smaller amounts in other marine foods [7]. Although more than 20 arsenosugars have been reported as natural products, most of the arsenic bound as arsenosugars is associated with just four compounds [7, 10, 11]. These arsenosugars are 5-deoxy-5-arsinoyl-β-d-riboside derivatives with variable side chain substitution at the C1 position, namely glycerol (—CH_2_CHOHCH_2_OH), glycerylsulfate (—CH_2_CHOHCH_2_OSO_3_H), glycerylsulfonate (—CH_2_CHOHCH_2_SO_3_H), or glycerylphosphate diester (—CH_2_CHOHCH_2_OP(O)(OH)OCH_2_CHOHCH_2_OH) resi-dues [12].

A risk assessment of arsenic in foods must be based not only on the type and quantities of the arsenicals present, but also on the metabolism of these compounds in humans. In the case of inorganic arsenic ingestion, arsenate can be reduced to arsenite, which can then be further transformed through a series of reductive methylation and conjugation reactions, some of which involve re-oxidation of trivalent to pentavalent arsenic [13]; these processes are believed to take place primarily in the liver. In cultured cells, some of the inorganic arsenic metabolites, including the trivalent mono- and dimethylated arsenicals (e.g. [14–21]) as well as thio-dimethylarsinic acid (thio-DMA^V^) (e.g. [22, 23]) have been shown to exert higher cytotoxicity and genotoxicity than arsenite. The major inorganic arsenic metabolite, dimethylarsinic acid (DMA^V^), exerts generally lower cellular toxicity but, in contrast to all other arsenicals tested so far, has been shown to be a complete carcinogen to the rat bladder [24, 25]. Data from epidemiological studies suggest that biomethylation of inorganic arsenic contributes to inorganic arsenic-induced carcinogenicity (e.g. [26–28]).

In contrast to the numerous studies on inorganic arsenic, there have been relatively few human metabolism studies on the organoarsenicals present in fish and seafood. Although arsenobetaine is bioavailable to humans, it is not metabolized and is rapidly excreted unchanged in urine [29]. Consequently, arsenobetaine is widely assumed to be of no toxicological concern [30–33]. In contrast, arsenosugars are metabolized by humans to a multitude of arsenic metabolites, which are subsequently excreted in the urine. Thus, after a single ingestion of synthetic 2′,3′-dihydroxypropyl 5-deoxy-5-dimethylarsinoyl-*ß*-d-riboside (DMA^V^-sugar-glycerol, AsS I) humans efficiently metabolized this arsenosugar [8, 34, 35]. Thereby, the excretion patterns indicated a strong individual variability in human metabolism [8, 35]. In human urine, traces of the intact DMA^V^-sugar-glycerol and more than ten metabolites were detected, including DMA^V^, the major metabolite, oxo-dimethylarsenoacetic acid (oxo-DMAA^V^), thio-dimethylarsenoacetic acid (thio-DMAA^V^), oxo-dimethylarsenoethanol (oxo-DMAE^V^), thio-dimethylarsenoethanol (thio-DMAE^V^), and thio-DMA^V^ [34, 35]. Additionally, thio-DMAA^V^ and thio-DMAE^V^ were detected in blood serum. Besides humans also sheep chronically exposed to arsenosugars via seaweed consumption seem to efficiently metabolize arsenosugars [36].

Regarding the in vivo toxicity of naturally occurring arsenosugars, there is only one very recent paper available indicating the induction of oxidative stress, DNA damage, and neurobehavioral impairments in mice after high-dose DMA^V^-sugar-glycerol ingestion (≥20 mg/kg b.w. and day) [37]. In vitro studies showed that DMA^V^-sugar-glycerol exerted no toxic effect up to the millimolar concentration range [38–40]. An IC_50_ value > 6 mM, determined by the neutral red uptake assay, was obtained for DMA^V^-sugar-glycerol in human keratinocytes; genotoxic potential was not observed in a DNA nicking assay nor was it observed in the Ames test. [40]. Likewise, cytotoxicity as measured by the Alamar Blue test occurred only in millimolar concentrations in BALB/c 3T3 mouse fibroblast cells and murine alveolar macrophages (IC_50_ = 6 mM, IC_50_ = 8 mM, respectively) [39]. Oya-Ohta et al. observed a significant increase of chromosomal aberrations in human fibroblasts after 24-h incubation with 12 mM DMA^V^-sugar-glycerol [38].

**Figure 1:**
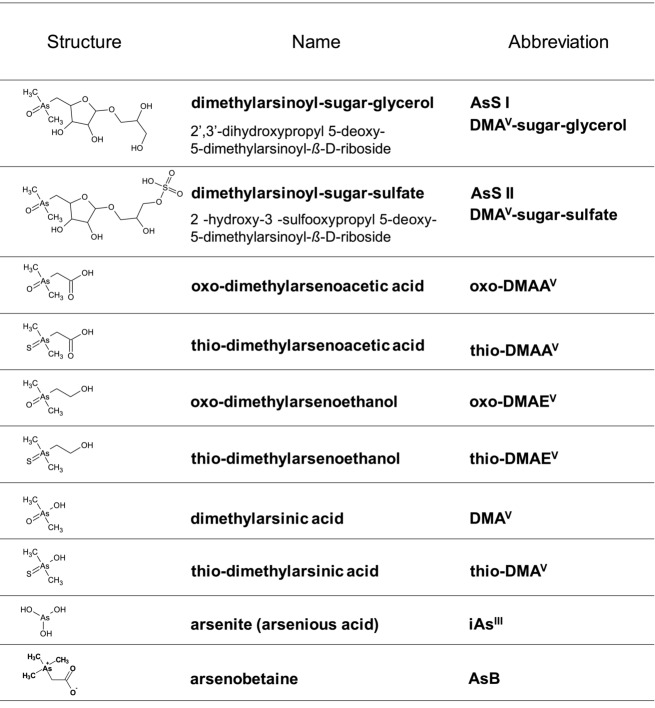
Chemical structures, names and abbreviations of ten arsenic species investigated in this study.

In their recently published Scientific Opinion on Arsenic in Food, the European Food Safety Authority Panel on Contaminants in the Food Chain concluded that a risk assessment for arsenosugars is currently not possible, largely because of the lack of relevant toxicological data. To address this issue, we report an extensive toxicological characterization of two arsenosugars (DMA^V^-sugar-glycerol and DMA^V^-sugar-sulfate) and their metabolites DMA^V^, thio-DMA^V^, oxo-DMAA^V^, thio-DMAA^V^, oxo-DMAE^V^, and thio-DMAE^V^ (Fig. 1) in cultured human bladder cells. Moreover, for the first time intestinal bioavailability of the arsenosugars was assessed applying the Caco-2 intestinal barrier model.

## 2 Material and methods

### 2.1 Caution

Inorganic arsenic is classified as a human carcinogen. The following chemicals are hazardous or potentially hazardous and should be handled with care: sodium (meta)-arsenite (iAs^III^), DMA^V^-sugar-glycerol (AsS I), DMA^V^-sugar-sulfate (AsS II), oxo-DMAA^V^, oxo-DMAE^V^, thio-DMAA^V^, thio-DMAE^V^, DMA^V^ and thio-DMA^V^.

### 2.2 Materials

Trypsin, fetal calf serum (FCS) and penicillin-streptomycin solutions were from PAA (Cölbe, Germany) and neutral red dye was supplied by Sigma-Aldrich (Steinheim, Germany). Minimum essential medium Eagle (MEM), nonessential amino acids and the culture dishes were obtained from Biochrom (Berlin, Germany). iAs^III^ (≥99% purity) and Alcian Blue were from Fluka Biochemika (Buchs, Germany), Transwell® filters were from Corning (Wiesbaden, Germany) and DMA^V^ (≥99% purity) was from Sigma-Aldrich. Giemsa dye, acridine orange, and formaldehyde were bought from Roth (Karlsruhe, Germany). Cell counting kit-8® was obtained from Dojindo Molecular Technologies, Germany. Hydrogen peroxide solution (30%, Suprapur) and nitric acid (65%, Suprapur) were products of Merck (Darmstadt, Germany). The ICPMS elemental standard (As, 1 mg/L) was purchased from SPETEC (Erding, Germany). All other proanalysis chemicals were obtained from Sigma-Aldrich or Merck.

The urothelial cell line, also known as human urothelial cells (UROtsa), was derived from a primary culture of a normal human urothelium through immortalization with the SV-40 large T antigen. This cell line was kindly provided by Professor M. Stýblo (University of North Carolina, USA). Caco-2 cells were obtained from the European Collection of Cell Cultures (ECACC; number 86010202, Salisbury, UK).

### 2.3 Synthesis, analysis and purity control of the arsenic species

Thio-DMA^V^ (>98%) was synthesized and purified as published before [23]. The following arsenicals were synthesized in Graz using published procedures: DMA^V^-sugar-glycerol, AsS I and DMA^V^-sugar-sulfate, AsS II (as the ammonium salt) according to Traar et al. [41]; arsenobetaine according to Cannon et al. [30]; oxo-DMAA^V^ according to Francesconi et al. [42]; oxo-DMAE^V^ by the method of Edmonds et al. [43], and thio-DMAA^V^ and thio-DMAE^V^ as reported by Raml et al. [34] The compounds were characterized and their purity was established by ^1^H and ^13^C NMR spectroscopy [30, 34, 41–43], molecular mass spectrometry (electrospray ionization; single quadrupole mass spectrometer, Agilent Technologies, Waldbronn), and HPLC/ICPMS (Agilent 1100 LC system coupled to an Agilent 7500ce ICPMS). When purity was established, each of the compounds was treated as follows. A portion (10–20 mg) of the arsenical was dissolved in ca. 10 mL water (Milli-Q, resistivity of 18.2 MΩ·cm, Millipore GmbH Vienna), and the solution was checked for arsenic concentration by ICPMS and for “arsenic species purity” by HPLC/ICPMS, which was >99.5% in all cases. Occasionally traces of DMA^V^, a byproduct from the reaction, were detected; inorganic arsenic was not detectable. Appropriate aliquots of this “stock” solution were transferred to Eppendorf tubes so that the quantity of arsenic in each tube was 250 μg As (= 3.33 μmoles of compound). A subset of these tubes was sent to the University of Muenster for cytotoxicity testing; the remaining tubes were held in Graz where the arsenicals were to serve as standards for later speciation analysis of metabolites.

### 2.4 Cell culture and incubation with the arsenicals

UROtsa were used as the in vitro model to study cellular toxicity, since both arsenosugars and their metabolites have been identified in human urine. Additionally, the bladder is an important target organ for inorganic arsenic-induced carcinogenicity in humans. UROtsa cells were cultured as a monolayer in MEM supplemented with FCS (10%, v/v), penicillin (100 U/mL) and streptomycin (100 μg/mL). The cultures were incubated at 37°C with 5% CO_2_ in air and 100% humidity. For each experiment, UROtsa cells were seeded in a defined density (17 000 cells cm^−2^). After 24 h, logarithmically growing UROtsa cells were incubated with the arsenic species for 48 h, as described for the respective experiments.

Caco-2 cells were grown as a monolayer in culture dishes in MEM supplemented with FCS (10%, v/v), nonessential amino acids (1%, v/v), glutamine (2 mM), penicillin (100 U/mL) and streptomycin (100 μg/mL). The cultures were incubated at 37°C with 5% CO_2_ in air and 100% humidity. Caco-2 cells were used for transfer experiments as described in 2.8.

Stock solutions of the respective arsenic species were prepared in sterile deionized water shortly before each experiment. The maximum concentration tested for each species was 500 μM; higher concentrations are unlikely to be exposure relevant.

### 2.5 Cytotoxicity testing

Cytotoxicity of the arsenic species was elucidated after 48-h incubation by quantifying their effect on cell number and colony-forming ability in a concentration range up to 500 μM. Additionally, cellular dehydrogenase activity and lysosomale integrity were assessed, applying the cell counting kit-8® (CCK-8®) and the neutral red uptake test.

Cell number and colony-forming ability testing were performed as described before [21, 23] with minor modifications. Briefly, after 48 h of incubation with the respective arsenicals, cells were washed with PBS and trypsinized. Cell number and cell volume were measured by an automatic cell counter CASY-TTC® (Roche Innovatis AG, Germany). These measurements are based on noninvasive (dye free) electrical current exclusion with signal evaluation via pulse area analysis. To assess the impact of the arsenic species on colony-forming ability, after cell counting of each sample, 500 cells/dish were seeded and cultivated. After 7 days, colonies were washed with PBS, fixed with ethanol, stained with Giemsa (25% in water), counted, and calculated as percent of control. The CCK-8® test and the neutral red uptake test represent well-accepted test systems to assess cell viability, and were performed as described before [23, 44].

### 2.6 Cellular bioavailability of the respective arsenicals

Briefly, logarithmically growing cells were exposed to the respective arsenic species for 48 h, trypsinized, collected by centrifugation, washed with ice-cold PBS, and cell number and cell volume were measured by an automatic cell counter (CASY-TTC®) in each sample. After incubation with the ashing mixture (65% HNO_3_/30% H_2_O_2_ (1:1, v/v)) at 95°C for at least 12 h and subsequent dilution with deionized water, arsenic was measured by electrothermal atomic absorption spectrometry (AAS; AAnalyst 600, PerkinElmer) as described before [23].

### 2.7 Formation of micronuclei, bi- and multinucleated cells

To investigate the induction of micronuclei, UROtsa cells were seeded in 12-well plates on Alcian blue-coated glass coverslips. After 24 h, cells were incubated with the respective arsenicals for 48 h, fixed with an ice-cold fixation solution (90% methanol/10% PBS, −20°C) for 10 min, dried in air at room temperature, stained with acridine orange (125 mg/L in PBS) for 60 s, and finally analyzed by fluorescence microscopy. Per coverslip, at least 1000 mononucleated cells were counted and categorized in mononucleated, binucleated, and multinucleated cells as well as cells with and without micronuclei; analyses were carried out after coding of slides.

### 2.8 Effects on and transfer across the Caco-2 intestinal barrier model

The human intestinal Caco-2 cell line is widely used as a model for the intestinal barrier [45, 46] and has been used to study transfer of arsenicals before [47–54].

Before transfer studies, cells were thawed and subcultured three times to achieve stable parameters in all experiments. For transfer experiments, Caco-2 cells (5 × 10^4^ cells cm^−2^) were seeded on Transwell® filter inserts with microporous polycarbonate membranes (1.12 cm^2^ growth area, 0.4 μm pore size) adding 0.5 mL culture medium to the apical and 1 mL culture medium to the basolateral compartment. After 11 days, cells were fully differentiated; during the differentiation process, culture medium was replaced every 2–3 days. The transepithelial electrical resistance (TEER) of the Caco-2 monolayer was used as a parameter for barrier integrity, measured by the cellZscope® (nanoAnalytics, Münster, Germany) device with a module suitable for a 24 Transwell® filter system. The determined capacitance is directly proportional to the plasma membrane surface area; changes in the basolateral area and changes in protein content and distribution may also contribute to capacitance changes. Only wells with TEER values > 1000 Ω × cm^2^ and capacitance values between about 3.8 to 5.0 μF cm^−2^ indicate a confluent monolayer with good barrier properties, and these wells were used for the respective experiments. All experiments were carried out with cells in passage number 53 on day 11 after initial seeding.

To study the impact of the arsenic species on the barrier integrity as well as their transfer across the in vitro barrier, the differentiated Caco-2 monolayer was exposed on day 11 to arsenic species on the apical side (referring to the intestinal lumen in vivo). During the following 48 h of incubation (thereby mimicking a continued exposure toward the arsenic species), aliquots were sampled from the apical and basolateral compartments for arsenic quantification by AAS, while online monitoring the respective TEER and capacitance values. Arsenic transfer from the apical to the basolateral compartment was expressed as percent permeability in relation to the applied concentration. Furthermore, total arsenic concentration in the acceptor compartment was shown. In the case of time-dependent linear correlation, permeability coefficients were calculated. The apparent permeability coefficient (P_c_) was calculated by applying Eq. (1) and (2).


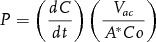
(1)


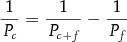
(2)


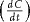
 is the concentration change (μM/s) determined by the linear slope of the concentration in acceptor compartment versus time plot. *V_ac_* is the volume of the acceptor compartment (basolateral: 1 mL). *A* is the growth area of the Transwell® (1.12 cm^2^) and *C_o_* is the initial concentration of the respective arsenic species in the donor compartment (500 μM of the arsenosugars and arsenobetaine, 1 μM iAs^III^). *P_f_* is the arsenic permeability of the Transwell® filter, *P_c+f_* the arsenic permeability of the filter in combination with the cell layer, and P_c_ the arsenic permeability of the cell layer only (Eq. (2)).

All studies were conducted in triplicate with independent cultures.

## 3 Results

### 3.1 Cytotoxicity

**Figure 2:**
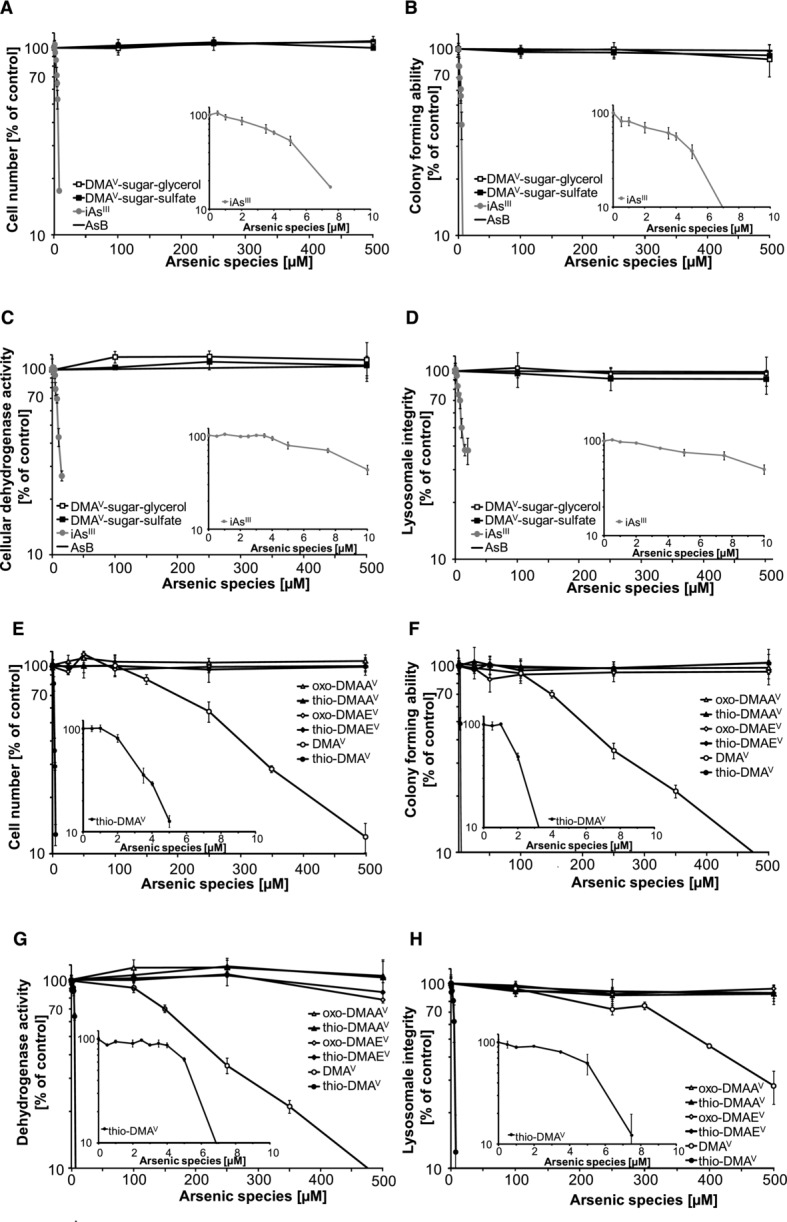
Cytotoxicity of DMA^V^-sugar-glycerol, DMA^V^-sugar-sulfate, arsenobetaine, iAs^III^ (A–D) as well as DMA^V^, thio-DMA^V^, oxo-DMAA^V^, thio-DMAA^V^, oxo-DMAE^V^ and thio-DMAE^V^ (E–H) in UROtsa cells after 48-h incubation. Cytotoxicity was determined by impact on cell number (A, E), colony-forming ability (B, F), dehydrogenase activity as measured by CCK-8® (C, G), and lysosomal integrity as measured by neutral red uptake (D, H). The data represent mean values of at least three determinations ± SD. Colony-forming ability of untreated control cells was approximately 80%.

For all endpoints, arsenobetaine, the two arsenosugars (Fig. 2A–D) and four metabolites, namely oxo-DMAA^V^, thio-DMAA^V^, oxo-DMAE^V^, and thio-DMAE^V^ (Fig. 2E–H), exerted no cytotoxicity after 48-h incubation at exposures up to 500 μM. In contrast, DMA^V^, thio-DMA^V^ (Fig. 2E–H), and arsenite (Fig. 2A–D) induced clear cytotoxicity in UROtsa cells. Thio-DMA^V^ and arsenite showed cytotoxic effects in a similar concentration range, with thio-DMA^V^ showing slightly stronger effects, whereas cytotoxicity from DMA^V^ was first observed at about 100-fold higher incubation concentrations (Table 1). For all three arsenic species, colony-forming ability was the most sensitive endpoint.

**Table 1:** Comparison of the investigated cytotoxicity endpoints after 48-h incubation with arsenite, DMA^V^, or thio-DMA^V^. Shown are the respective concentrations of the arsenic species, causing a 30% reduction in cell number, colony-forming ability, dehydrogenase activity and lysosomale integrity, respectively

30% reduction in	DMA^V^	thio-DMA^V^	iAs^III^
Cell number	205 μM	2.3 μM	3.6 μM
Colony-forming ability	150 μM	1.5 μM	2.0 μM
Dehydrogenase activity	249 μM	4.7 μM	5.7 μM
Lysosomale integrity	319 μM	4.3 μM	7.5 μM

### 3.2 Cellular bioavailability

**Figure 3:**
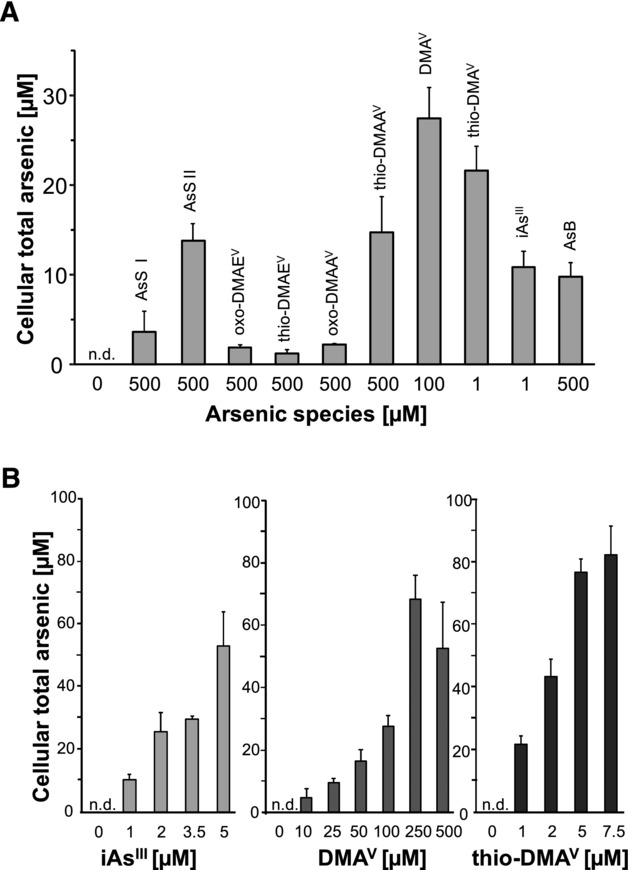
Cellular bioavailability of the arsenicals after 48-h incubation of UROtsa cells (A). Concentration-dependent bioavailability after 48-h incubation with iAs^III^, DMA^V^, and thio-DMA^V^ (B). The figure represents mean values of at least three independent determinations + SD; n.d. below LOD.

To assess cellular bioavailability and to correlate cellular toxicity of the respective arsenic species with cellular total arsenic content in UROtsa cells, cellular arsenic concentrations were determined after 48-h incubation. The determined cellular total arsenic concentrations clearly show that all investigated arsenicals were bioavailable to UROtsa cells (Fig. 3A).

Comparing the extracellular arsenic concentration with the measured cellular concentration, thio-DMA^V^ and arsenite were accumulated by the cells by a factor of about 22- to 10-fold (Fig. 3B). In case of DMA^V^, about 100-fold higher incubation concentrations were necessary to achieve total cellular arsenic concentrations comparable to those occurring after 48-h incubation of thio-DMA^V^ and arsenite. However, total cellular arsenic concentrations were still higher after incubation with DMA^V^ than in the experiments with the arsenosugars and their larger metabolites.

### 3.3 Formation of micronuclei, bi and multinucleated cells by the arsenicals

To assess the induction of micronuclei by the arsenicals, we omitted the application of cytochalasin B, since our earlier studies indicated that several arsenicals interact with actin and/or the effect of cytochalasin B [23]. To ensure mitosis, we controlled cell proliferation by means of cell number quantification and chose an incubation time of 48 h, which is equivalent in UROtsa cells to 2–2.2 cell cycles of untreated control cells.

**Figure 4:**
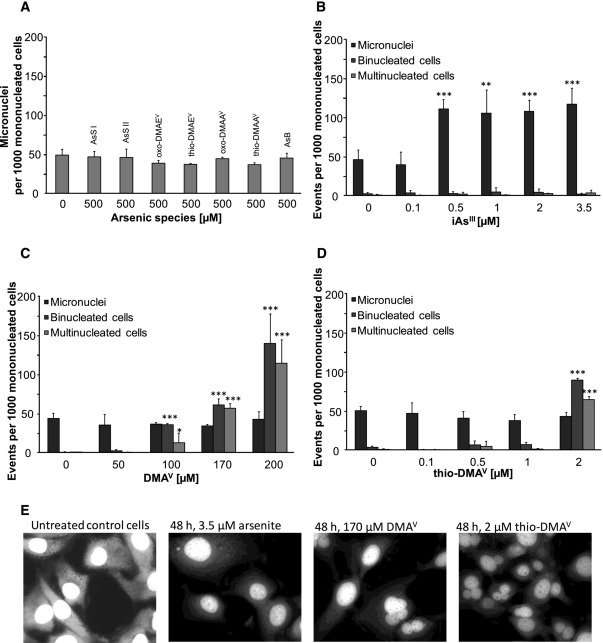
Formation of micronuclei in UROtsa cells after 48-h incubation with DMA^V^-sugar-glycerol, DMA^V^-sugar-sulfate, oxo-DMAA^V^, thio-DMAA^V^, oxo-DMAE^V^, thio-DMAE^V^ or arsenobetaine (A). Increase in the number of micronuclei, bi- and multinucleated UROtsa cells after 48-h incubation with arsenite (B), DMA^V^ (C), or thio-DMA^V^ (D). Displayed are mean values of at least three independent determinations + SD. Representative fluorescence microscopic images (E). Asterisks mark statistically significant differences as compared to untreated cells (*, *p* < 0.05; ^**^, *p* < 0.01; ^***^, *p* < 0.001).

For arsenobetaine, the two arsenosugars and the four metabolites oxo-DMAA^V^, thio-DMAA^V^, oxo-DMAE^V^, and thio-DMAE^V^, no induction of micronuclei at the applied dose was observed (Fig. 4A). Similarly, no increase in the number of bi- or multinucleated cells occurred for these arsenic species (data not shown). At incipient cytotoxic concentrations, DMA^V^ (Fig. 4C and E) and thio-DMA^V^ (Fig. 4D and E) exposure led to significant increases in multinucleated cells and binucleated cells in comparison to untreated control cells. However, both DMA^V^ and thio-DMA^V^ showed no micronuclei induction. In contrast, iAs^III^ induced micronuclei formation even in the subcytotoxic concentration range, but did not result in an increase in the number of bi- or multinucleated cells (Fig. 4B and E).

### 3.4 Effects of the arsenosugars on and transfer across the Caco-2 intestinal barrier model

Intestinal bioavailability of the arsenosugars in relation to arsenite and arsenobetaine was assessed by measuring their time-dependent crossover after apical incubation in the Caco-2 barrier model, while online monitoring of barrier integrity. Here the differentiated Caco-2 cells grown on Transwell® filters build a two-chamber model, with the cell layer resembling the intestinal barrier, the upper apical side referring to the intestinal lumen, and the lower basolateral side referring to the blood side.

**Figure 5:**
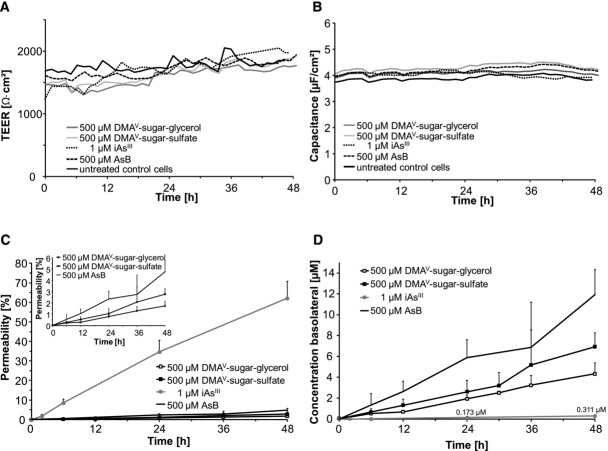
Effects of the arsenicals on and transfer across the intestinal Caco-2 barrier model after apical incubation. Transepithelial electrical resistance (TEER) (A) and capacitance (B) were used as tracers for barrier integrity; shown are representative measurements. Arsenic concentration in the acceptor (basolateral) compartment (C), percent arsenic permeability (normalized to the respective incubation concentration) (D). Shown are mean values of at least three independent determinations +SD.

During 48 h of incubation, neither 500 μM DMA^V^-sugar-glycerol nor 500 μM DMA^V^-sugar-sulfate affected barrier integrity or cell vitality as measured by TEER (Fig. 5A) and capacitance (Fig. 5B) values, respectively. Additionally, the two reference compounds, arsenite and arsenobetaine, were tested at the same time to check comparability with published data. Neither arsenic species impacted the barrier integrity at the applied concentration (Fig. 5A and B).

Whereas arsenite crossover was very efficient, the transfer rates of the organoarsenicals were quite small. After 48-h apical incubation with 500 μM of DMA^V^-sugar-glycerol, DMA^V^-sugar-sulfate or arsenobetaine, in the acceptor compartment 4.33 ± 1.07 μM, 6.94 ± 1.35 μM, 11.93 ± 2.41 μM arsenic were measured, respectively (Fig. 5D). Taking into account the twofold higher volume in the basolateral compartment, this refers to a permeability of 1.7 ± 0.4, 2.8 ± 0.5, 4.8 ± 1.0% for DMA^V^-sugar-glycerol, DMA^V^—sugar-sulfate or arsenobetaine, respectively (Fig. 5C). In the case of 48-h incubation with 1 μM arsenite, 0.31 ± 0.04 μM was measured in the acceptor compartment, referring to a permeability of 62.1 ± 8.7% (Fig. 5C and D). To better compare our data with literature data, permeability coefficients were also calculated (Table 2).

**Table 2:** Permeability coefficients for the respective arsenicals after apical incubation. Shown are mean values of at least three independent determinations ± SD; a, apical; b, basolateral

Permeability coefficient (a → b; after 24 h)	
DMA^v^-sugar-glycerol (500 μM)	5.1 × 10^−8^ ± 1.3 × 10^−8^
DMAv-sugar-sulfate (500 μM)	7.1 × 10^−8^ ± 1.3 × 10^−8^
iAs^III^ (1 μM)	1.6 × 10^−6^ ± 0.5 × 10^−6^
AsB (500 μM)	11.6 × 10^−8^ ± 2.8 × 10^−8^

## 4 Discussion

This study investigated the cellular toxicity and assessed the intestinal bioavailability of two arsenosugars naturally present in marine foods. For the first time, cellular toxicity of the arsenosugars was studied in direct comparison to the effects of six arsenosugar metabolites as well as the reference substances, first being arsenite (iAs^III^), which is classified as a human carcinogen [1], and second being arsenobetaine, which is known to be harmless to humans [4].

In the applied concentration range, up to 500 μM the two arsenosugars, DMA^V^-sugar-glycerol and DMA^V^-sugar-sulfate, as well as arsenobetaine exerted neither cytotoxicity nor genotoxicity, even though they were bioavailable to UROtsa cells. Regarding DMA^V^-sugar-glycerol, the observed lack of cellular toxicity in the micromolar concentration range is in accordance with earlier studies [38–40]. Additionally, our data indicate that this lack of toxicity is not due to low cellular bioavailability of arsenosugars. In direct comparison to DMA^V^-sugar-glycerol, DMA^V^-sugar-sulfate, which has never been toxicologically characterized before, shows about a fourfold higher bioavailability to UROtsa cells. The trivalent arsenosugar analogue DMA^III^-sugar-glycerol has been reported to show cellular toxicity in cultured human keratinocytes [40], however the existence of this arsenical in biological samples is yet to be analytically proven.

DMA^V^ has been identified as the major human urinary metabolite after both inorganic arsenic and DMA^V^-sugar-glycerol intake; moreover, thio-DMA^V^ has been identified as a common metabolite. In contrast, oxo-DMAA^V^, thio-DMAA^V^, oxo-DMAE^V^, and thio-DMAE^V^ are exclusive metabolites of arsenosugars [8, 34, 35, 55]. In the present study, these four arsenosugar metabolites behaved similarly to the parent arsenosugars – they were bioavailable to the cells but did not show cellular toxicity in any of the investigated cytotoxicity and genotoxicity endpoints. With respect to effects on cellular dehydrogenase activity, this lack of toxicity is in accordance with data published in cultured human hepatocarcinoma (HepG2) cells applying the WST-8 test [34]. Other cytotoxicity endpoints, cellular bioavailability and genotoxicity of oxo-DMAA^V^, thio-DMAA^V^, oxo-DMAE^V^ and thio-DMAE^V^ have not been studied before. A direct comparison of the effects of the organoarsenicals with arsenite in this study clearly indicates that cellular toxicity is not only related to the cellular total arsenic concentration but also depends on the arsenic species incubated. Thus, 48-h incubation with 500 μM DMA^V^-sugar-sulfate, arsenobetaine or thio-DMAA^V^ resulted in a similar or even higher cellular total arsenic content than 48-h incubation with 1 μM arsenite. Nevertheless, incubation with just 1 μM arsenite caused a decrease in colony-forming ability as well as a strong increase in micronuclei frequency.

The common inorganic arsenic and arsenosugar metabolites DMA^V^ and thio-DMA^V^ caused moderate and strong cellular toxicity in UROtsa cells, respectively. Depending on the endpoint studied, thio-DMA^V^ exerted up to 100-fold higher cytotoxicity than DMA^V^; indeed, thio-DMA^V^ was slightly more cytotoxic than arsenite. In terms of cytotoxicity, this order of the arsenicals has been shown before in cultured human lung, liver, and bladder cells [22, 23], and is strongly related to the cellular bioavailability of these arsenicals. For all three arsenicals, colony-forming ability turned out to be the most sensitive cytotoxicity endpoint investigated, suggesting an indirect mode of toxic action for these arsenicals.

As previously reported (e.g. [23, 56, 57]), our reference compound arsenite induced micronuclei, whereas both DMA^V^ and thio-DMA^V^ increased the frequency of bi- and multinucleated cells, indicating cell-cycle arrest and disturbance in mitosis. On the cellular level, thio-DMA^V^ and DMA^V^ seem to exhibit genotoxicity in a similar manner, which is most likely different from that shown by arsenite. When additionally taking into account cellular bioavailability of the arsenic species, effects induced after thio-DMA^V^ and DMA^V^ incubation occur at comparable total cellular arsenic concentrations. These facts suggest that after incubation with either thio-DMA^V^ or DMA^V^, a common arsenic species is formed inside the cell causing the respective cytotoxic and genotoxic effects. A possible candidate is DMA^III^, which has recently been discussed to be formed inside the cell from DMA^V^ and thio-DMA^V^ [58]. However, this assumption needs further experimental support including rigorous analytical confirmation. Genotoxic effects have been described for all three dimethylated metabolites, DMA^V^, DMA^III^ and thio-DMA^V^, with DMA^III^ often being the most potent species [23, 58–62].

When humans consume arsenosugars, whether present in seafood [33, 63, 64] or ingested as a pure synthesized compound [8, 34, 35], they efficiently metabolize these organoarsenicals. Six arsenosugar metabolites have been identified in humans so far, with DMA^V^ being the major metabolite. Moreover, traces of arsenosugars themselves have also been identified in human urine [8, 34, 35], indicating that at least a fraction of the ingested arsenosugars are absorbed in the form of intact arsenosugars via the gastrointestinal tract. In vitro simulation studies provided evidence that arsenosugars might be stable during stomach passage [65]. Regarding the rate of absorption of arsenosugars, there are conflicting data in the literature. In vitro digestion studies indicate a strong bioaccessibility (>80%) [66]. In an early in vivo study*,* approximately 80% of ingested DMA^V^-sugar-glycerol (equivalent to 1220 μg arsenic) was excreted in urine in one male volunteer during 4 days after ingestion [8], giving evidence of almost complete absorption in humans. However, recent data based on urinary excretion after single dose oral intake of these arsenosugars suggest considerable individual variability in the absorption and metabolism of arsenosugars [35]. Among the six volunteers, 4-98% of the ingested arsenic was excreted via urine within 4 days. Our data obtained from the Caco-2 intestinal bioavailability studies show a rather low transfer of the arsenosugars across the intestinal barrier model. Thereby, the applied sulfate arsenosugar was about 1.6-fold more bioavailable than the glycerol sugar that had been ingested by volunteers in earlier experiments [8, 34, 35]. Thus, within 48 h, 1.7 ± 0.4 and 2.8 ± 0.5% of the applied DMA^V^-sugar-glycerol and DMA^V^-sugar-sulfate crossed the intact Caco-2 barrier, probably via paracellular transfer. Nevertheless, paracellular transfer of the arsenosugars might be underestimated by the Caco-2 model. Paracellular transfer is often underestimated by the Caco-2 model because of the strong tightness of tight junctions in comparison to the in vivo situation [67]. In vivo tight junctions of the intestinal mucosa are much more permeable [68, 69]. The transfer rate of the arsenosugars was slightly lower than the crossover of arsenobetaine and much lower compared to the transfer rates of arsenite; the observed transfer rates of both arsenite and arsenobetaine are similar to the respective Caco-2 transfer rates that have been published before [47, 49, 51]. Together with the observed high total arsenic absorption of the ingested DMA^V^-sugar-glycerol in some volunteers, these data point to the possibility that arsenosugars are at least partly biotransformed before absorption. Accordingly, Conklin et al. demonstrated bioconversion of DMA^V^-sugar-sulfonate to its thio-analogue applying mouse cecal microflora and tissue [70]; also for other arsenicals presystemic metabolism by cecal tissue and microbial flora to higher methylated or thiolated arsenic species has been postulated [70–73]. Presystemic metabolism is likely to affect toxico Kinetics as well as toxicity of the arsenosugars and interindividual differences in presystemic metabolism may also contribute to the great individual variability in arsenosugar metabolism observed by Raml et al. [35].

Asian population groups in general consume comparably high amounts of seaweed, some of which contain more than 100 mg of arsenic/kg dry mass [10], with arsenosugars being the predominant arsenic fraction. In Japan, exposure to arsenosugars from seaweed consumption can be as high as 1 mg/day [74]. Furthermore, in the United States and Europe seaweed consumption, usage of marine algae-based food supplements, and food additives based on marine algae are increasing and therewith arsenosugar exposure. Additionally, the consumption of mussels and oysters might strongly contribute to arsenosugar intake. Immediately after the discovery of the metabolism of arsenosugars to DMA^V^ in sheep by the Feldmann group [75], concern was voiced that DMA^V^ might pose a risk to human health [76], particularly since DMA^V^ has been shown to be a complete carcinogen in the rat [24, 25]. These fears have been strengthened by the arsenosugar metabolism studies in humans, which clearly indicated that after arsenosugar intake, likewise after inorganic arsenic intake, DMA^V^ is the major (>50%) human urinary metabolite. To compare amounts of DMA^V^ formation after arsenosugar or inorganic arsenic intake by humans, a worst case scenario can be assumed, based on the volunteers that excreted up to 98% arsenic of the ingested arsenosugar dose [35]. When eating 10 g algae (dry weight) with a content of 25 mg arsenic/kg dry weight per day, an individual consumes approximately 250 μg arsenic mostly in the form of arsenosugars and might be expected to excrete approximately 120 μg DMA^V^. To reach similar amounts of DMA^V^ from inorganic arsenic intake would require consumption of ca. 20 L of water containing the maximum permissible arsenic content of 10 μg/L [31]. Arsenosugars might also show toxic effects via the formation of thio-DMA^V^, which exerts cellular toxicity in about 100-fold lower concentrations compared to DMA^V^, as shown in the present study. Moreover, thio-DMA^V^ has been shown to affect the cellular oxidative stress response in cultured human lungs cells in the ultralow, picomolar concentration range [77]. Additionally, the thio-analogues of the arsenosugars, which might be formed in the gastrointestinal tract [70], are likely to exert higher bioavailability and toxicity.

In summary, we demonstrate that the sulfate and glycerol arsenosugar investigated in this study, as well as four arsenic metabolites of these two arsenosugars, exert no cellular toxicity up to 500 μM exposure in cultured human bladder cells, even though they are bioavailable to the cells. However, DMA^V^ and in particular its S-analogue thio-DMA^V^ are toxic to bladder cells in the micromolar concentration range. Thus, it is likely that in a cellular system that metabolizes arsenosugars, especially to thiolated metabolites, cellular toxicity might arise. Moreover, arsenosugars are intestinally bioavailable, both in vivo [8, 34, 35] as well as in the Caco-2 model. Therefore, in strong contrast to arsenobetaine, arsenosugars cannot be categorized, as nontoxic for humans and a risk to human health cannot be excluded.

## Abbreviations

DMA^V^: dimethylarsinic acid
DMA^V^-sugar-glycerol, AsS I: 2′,3′-dihydroxypropyl 5-deoxy-5-dimethylarsinoyl-*ß*-d-riboside
DMA^V^-sugar-sulfate, AsS II: 2'-hydroxy-3'-sulfooxypropyl 5-deoxy-5-dimethylarsinoyl-*ß*-D-riboside
FCS: fetal calf serum
iAs^III^: sodium (meta) arsenite
oxo-DMAA^V^: oxo-dimethylarsenoacetic acid
oxo-DMAE^V^: oxo-dimethylarsenoethanol
thio-DMA^V^: thio-dimethylarsinic acid
thio-DMAA^V^: thio-dimethylarsenoacetic acid
thio-DMAE^V^: thio-dimethylarsenoethanol
TEER: transepithelial electrical resistance
UROtsa: human urothelial cells
